# Effect of Oral Nutritional Supplementation on Health-Related Outcomes and Nutritional Biomarkers in Children and Adolescents with Undernutrition: A Systematic Review and Meta-Analysis

**DOI:** 10.3390/nu16172970

**Published:** 2024-09-03

**Authors:** Ruopeng An, Jing Shen, Zhiying Zhang, Meng Thiam Lim, Dieu T. T. Huynh

**Affiliations:** 1 Brown School, Washington University in St. Louis, 1 Brookings Drive, St. Louis, MO 63130, USA; ruopeng@wustl.edu; 2 Department of Physical Education, China University of Geosciences (Beijing), No. 29, Xueyuan Road, Haidian District, Beijing 100083, China; shenjing@cugb.edu.cn; 3 Abbott Nutrition Research and Development Asia-Pacific Center, 20 Biopolis Way, 09-01/02 Centros Building, Singapore 138668, Singapore; zinnia.zhang23@gmail.com (Z.Z.); mengthiam.lim@abbott.com (M.T.L.)

**Keywords:** pediatric malnutrition, dietary supplementation, infections, biochemical indices

## Abstract

This systematic review aims to synthesize scientific evidence on the effects of oral nutritional supplementation (ONS) on health-related outcomes and nutritional biomarkers among children and adolescents with undernutrition. The review protocol was reported following the Preferred Reporting Items for Systematic Review and Meta-Analysis Protocols (PRISMA-P) guidelines. A comprehensive keyword and reference search was conducted in seven electronic bibliographic databases: PubMed, Academic Search Complete, Academic Search Premier, CINAHL, Global Health, Web of Science, and Scopus. We identified 14 peer-reviewed articles reporting results from 13 unique studies (eight randomized controlled trials, four pre-post studies, and one observational study). Study participants were recruited from 14 countries/regions, with ages ranging from 1 to 14 years. Outcomes of interest include health-related outcomes (acute diseases and infections) and nutritional biomarkers (e.g., serum iron and zinc). Six of the eight studies examining acute diseases/infections and five of the seven examining nutritional biomarkers reported statistically significant improvement in some, but not all, outcomes. A meta-analysis of three studies found that ONS interventions reduce the incidence of upper respiratory tract infection (URTI) by 39% (95% CI, 0.42–0.91) in children at nutritional risk when compared to dietary counseling (DC) alone. This systematic review suggests that ONS interventions can improve certain health-related outcomes and nutritional biomarkers in undernourished children and adolescents. Specifically, the use of ONS significantly reduces the risk of URTI, highlighting its potential to enhance immune function and break the cycle of undernutrition and infection.

## 1. Introduction

Undernutrition in children and adolescents, characterized by wasting, stunting, and being underweight, has been a global public health challenge with broad implications [1,2]. In 2022, it was estimated that 148 million children under the age of five were stunted, and 45 million were wasted globally [3]. Approximately half of the childhood deaths are attributed to undernutrition, according to UNICEF [4]. The medical, developmental, and socioeconomic impacts of the global burden of undernutrition are profound and long-lasting for children and adolescents, their families, local communities, and countries [5].

A two-way relationship, or a “vicious cycle” linking undernutrition to acute diseases/infections, has been extensively documented. Undernutrition elevates the susceptibility to acute diseases/infections, and acute diseases/infections aggravate undernutrition by suppressing appetite, inducing catabolism, and elevating demand for nutrients [6,7,8]. The increased vulnerability to acute diseases/infections is thought to be partly caused by impaired immune function due to undernutrition [9]. Undernutrition has significantly contributed to immunodeficiency, most pronounced among infants, children, adolescents, and older adults [10,11,12]. Deficiencies in protein-energy and micronutrients are linked to impairments in cell-mediated immunity, antibody levels, phagocytic activity, the complement system, and cytokine production [13].

In the wake of the novel coronavirus disease 2019 (COVID-19), the relationship between undernutrition and infections has become more relevant than ever [14]. Poor diet may compromise nutritional status, resulting in susceptibility to COVID-19 infections [15]. The United Nations Children’s Fund promotes three strategies for preventing and treating undernutrition in children and adolescents, including dietary diversification, food fortification, and micronutrient supplementation [16].

One of the key goals of nutrition intervention in treating undernourished children is to strengthen their resistance to infections [17]. Micronutrients, including zinc, selenium, iron, copper, β-carotene, and vitamins A, C, and E, as well as folic acid, can affect various aspects of innate immunity [18]. Nutrients also play a role in initiating and regulating adaptive immune responses by influencing the differentiation, proliferation, and activation of B and T lymphocytes, as well as antibody production [19]. In addition, prebiotics and probiotics may promote a healthy microbial composition and help modulate the host immune system [20,21,22].

Oral nutritional supplements (ONS) are sterile formulations available in liquid, semi-solid, or powdered forms, supplying both macronutrients and micronutrients [23]. ONS are frequently utilized in both acute care and community health settings for individuals unable to fulfill their nutritional needs through a standard diet alone [23]. ONS may benefit children and adolescents with growth faltering, selective diets, poor appetite, chronic diseases, and developmental disabilities [24]. Standard ONS are formulated according to dietary recommendations to support children’s growth. For example, the macronutrient distribution in ONS formulation usually follows the US Institute of Medicine’s (or local) recommendations for typical diets for children and adolescents where the energy contributions of protein, fat, and carbohydrate are 10–15%, 30–35%, and 50–60%, respectively [25]. In addition, the fatty acid composition and content of essential fatty acids in ONS are designed to meet the requirements for tissue incorporation and specific body functions in children and adolescents [26]. ONS typically have an energy density between 1.0–1.5 kcal/mL with a high nutrient concentration, which helps support a balanced accretion of lean and fat tissues and regain normal growth [27]. ONS are “nutritionally complete” because they contain all essential nutrients in proportions that make them suitable for use as a sole source of nutrition [28].

Zhang et al. conducted a systematic review and meta-analysis to assess the effectiveness of ONS interventions on growth outcomes among 9-month-to-12-year-old undernourished children [29]. Eleven randomized controlled trials (RCTs) were included [29]. ONS use was found to significantly improve various anthropometric measures (e.g., weight and height) compared to controls (e.g., under a usual diet, placebo, or dietary counseling alone) [29]. Consistent with this finding, a newly published RCT again demonstrated that adding ONS to dietary counselling (DC) resulted in better weight and height gain and linear catch-up growth in children with or at risk of undernutrition, compared to DC alone [30] A recent systematic review synthesized research on the application of ONS for children 1–18 years with or at risk of faltering growth, focusing on subjects recruited from clinical settings [31]. The review analyzed 10 RCTs involving 1116 children and found that the utilization of ONS resulted in significant enhancements in weight and height, likely a consequence of improved nutritional intake [31].

Considering the bi-directional relationship between nutrition and infections, we believe evaluating the effect of ONS intake on health-related outcomes is crucial. In the aforementioned systematic review, the researchers also investigated the effect of ONS on clinical outcomes and concluded that there is a possible association between ONS use and reduced infections [31]. However, it is noteworthy that meta-analysis was not conducted for any clinical outcome, and the publication’s search strategy only included studies up to November 2021.

Multiple nutrient deficiencies are common in children with or at risk of undernutrition. Therefore, the goals of nutrition interventions are to provide adequate nutrition to rectify these deficiencies and promote catch-up growth [17]. Since the consumption of ONS has been shown to promote catch-up in height among children with or at risk of undernutrition, the use of nutrient-dense ONS is presumed to have enhanced the nutrition quality of the children’s diet [17]. It would therefore be of interest to determine whether ONS intake can address nutrient deficiencies, as assessed by biomarker status.

Complementing previous reviews [29,31] that have reported improvements in anthropometric measures, our study aimed to comprehensively identify and synthesize the scientific evidence concerning the effects of ONS on health-related outcomes among children and adolescents with or at risk of undernutrition. Changes in nutritional biomarkers and other relevant biochemical indices were also summarized. Review findings may address a critical gap in the scientific literature and inform evidence-based practices and policies to prevent detrimental health outcomes among undernourished minors.

## 2. Methods

### 2.1. Review Protocol and Registration

The review protocol was designed based on the methodological framework outlined in the Cochrane Handbook for Systematic Reviews of Interventions and reported in accordance with the Preferred Reporting Items for Systematic Review and Meta-analysis Protocols (PRISMA-P) guidelines [32,33]. The protocol was registered in the PROSPERO database (Registration No. CRD42022292035) [34].

### 2.2. Study Selection Criteria

We adopted the PICOS framework (Population, Intervention, Comparison, Outcome, Study design) to guide our data collection and synthesis process [35]. Studies that fulfilled all of the following criteria were included in the review: (1) Study designs: human clinical trials (i.e., randomized, quasi-randomized, or non-randomized controlled trials, or single-arm trials with pre- vs. post-intervention comparisons) and observational studies; (2) Study participants: children or adolescents aged 1–19 years with mild, moderate, or severe undernutrition, who were either clinically healthy or had acute respiratory or gastrointestinal infections not requiring hospitalization; (3) Type of ONS: ONS containing at least one non-protein calorie source (carbohydrate or fat) and nitrogen (in the form of intact protein, hydrolyzed protein, or amino acids), along with a full spectrum of micronutrients; (4) Exposure: ONS administered orally, in any amount, and for a given period; (5) Health-related outcomes: number of sick days, rate of recurrent infections, acute health events (e.g., diarrhea, vomiting, nausea, constipation, and allergies), comorbidity, or mortality; (6) Nutritional biomarkers and relevant biochemical indices such as serum iron, zinc, albumin, total protein, hemoglobin, ferritin, calcium, and phosphorus; (7) Type of article: original, empirical studies published in peer-reviewed journals; (8) Search timeframe: from the database’s inception to 1 February 2023; and (9) Language: articles published in English.

Studies meeting any of the following criteria were excluded from this review: (1) Animal or cell-culture studies; (2) Infant (younger than one-year-old) or adult (20 years and above) participants; (3) Children or adolescents without undernutrition; (4) Children or adolescents with pre-existing chronic diseases such as cystic fibrosis, HIV/AIDS, or cancer; (5) ONS that contain only, or predominantly, a single macronutrient (fat, carbohydrate, or protein), lipid-based supplements, or micronutrient-only (vitamins or minerals) supplements; (6) Therapeutic food products; (7) Semi-elemental formulations or fortified milk; (8) Studies of enteral tube feeding or parenteral nutrition; and (9) Letters, editorials, study or review protocols, case reports, or review articles.

The World Health Organization (WHO) has developed various childhood growth standards by age and sex [36]. The three most widely adopted anthropometric measures are weight-for-age, height-for-age, and weight-for-height, which can be expressed as *z*-scores or percentiles [36]. Other measures, including body mass index (BMI)-for-age and mid-arm circumference-for-age, are also used to assess growth [36]. The risk of undernutrition can be classified as mild (−2 ≤ *z*-score < −1), moderate (−3 ≤ *z*-score < −2), and severe (*z*-score < −3) [37]. Stunting is defined as a height-for-age *z*-score < −2, wasting as a weight-for-height *z*-score < −2, and underweight as a weight-for-age *z*-score < −2 [38].

### 2.3. Search Strategy

A keyword search was conducted across seven electronic bibliographic databases: PubMed/MEDLINE, Academic Search Complete, Academic Search Premier, CINAHL, Global Health, Web of Science (including Science Citation Index Expanded, Social Sciences Citation Index, and Emerging Sources Citation Index), and Scopus. The search algorithm includes keywords concerning ONS (e.g., “oral nutritional supplement”, “protein-energy supplement”), children/adolescents, and undernutrition (e.g., “under-weight”, “wasting”, or “stunting”). The search algorithm (Appendix A) was used to identify relevant titles and abstracts in the databases. Two co-authors independently screened the titles and abstracts found through the keyword search, retrieved articles that appeared eligible, and reviewed their full texts. Inter-rater agreement between the two co-authors was evaluated using Cohen’s kappa (κ = 0.82). Any discrepancies were resolved through consultation with a third co-author.

A backward reference search and a forward citation search were performed using the full-text articles identified through the keyword search that satisfied the study selection criteria. Articles retrieved from both the backward and forward searches were subsequently screened and evaluated using the same selection criteria. This process was repeated for all newly identified articles until no further relevant articles were found.

### 2.4. Data Extraction and Synthesis

A standardized data extraction form was utilized to gather the following methodological and outcome variables from each included study: author(s), year of publication, country, participants’ undernutrition status, participants’ mean age and age range, participants’ sex distribution, study design, sample size, intervention setting, intervention duration, arm-specific sample size, arm-specific intervention assignment, characteristics of the ONS product(s) used in the intervention, ONS daily dose, health-related outcomes, nutritional biomarkers, and intervention effectiveness (i.e., outcome-specific treatment effect estimates).

### 2.5. Meta-Analysis

The eligibility criterion for a meta-analysis is two or more studies of the same study design (e.g., RCT) assessing the same measure (e.g., number of sick days), with relevant treatment effect and standard error reported (or allowing the calculation of these two through the provision of relevant statistics, such as mean outcome measures in the treatment and control arms together with their respective standard deviations). All health-related outcomes and nutritional biomarkers reported in the included studies were assessed for their meta-analysis feasibility. The only eligible outcome identified is the incidence of upper respiratory tract infection (URTI). We used the *I*^2^ index to assess heterogeneity across studies eligible for meta-analysis. The *I*^2^ index indicates the proportion of variability in effect estimates among studies that is attributable to heterogeneity rather than random chance [32,39]. An *I*^2^ index greater than zero indicates the presence of heterogeneity, and an *I*^2^ index over 50% indicates a high level of heterogeneity [32,39]. A zero or low level of heterogeneity suggests a fixed-effect model, whereas a high level of heterogeneity supports a random-effect model [32,39]. A fixed-effect model was estimated because the calculated *I*^2^ index for the URTI outcome approximates zero. The meta-analysis was conducted using R version 4.2.

### 2.6. Study Quality Assessment

The Grading of Recommendations, Assessment, Development, and Evaluations (GRADE) framework is employed for creating and presenting evidence summaries and offers a systematic approach to formulating clinical practice recommendations [40]. GRADE assesses each study and assigns it to one of four evidence levels: very low, low, moderate, or high. Randomized controlled trials (RCTs) begin with a high evidence rating, while observational studies typically start at a low evidence rating due to residual confounding [40]. The quality of evidence for a study can be adjusted up or down during evaluation based on GRADE criteria, which consider factors such as risk of bias, imprecision, inconsistency, indirectness, and publication bias [40].

## 3. Results

### 3.1. Identification of Studies

**Figure 1:**
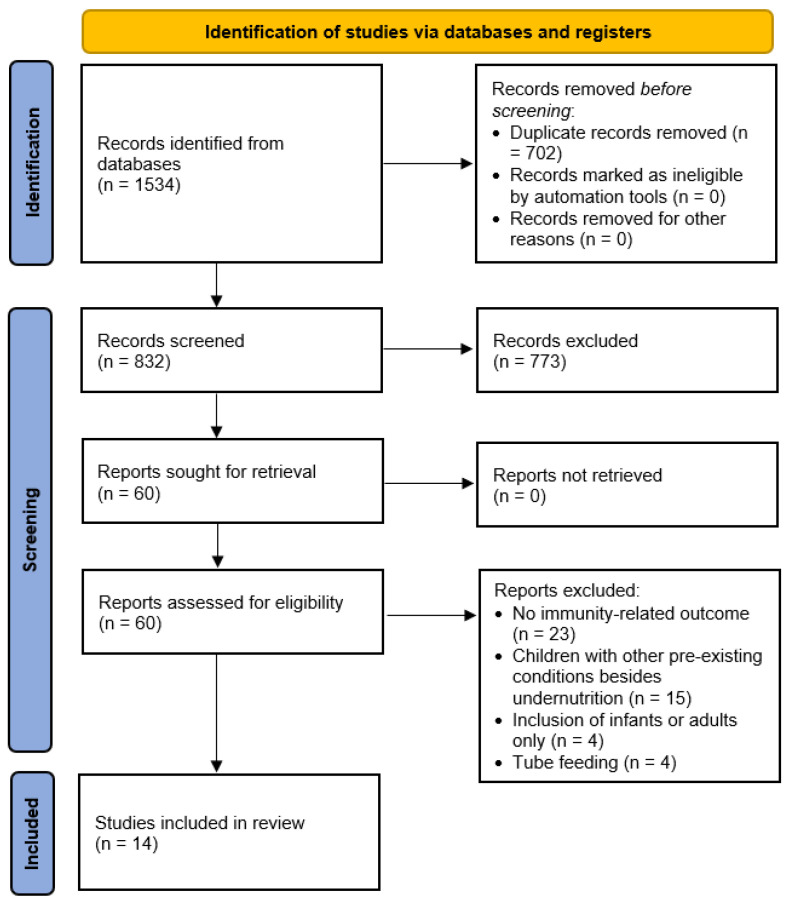
PRISMA 2020 flow diagram.

Figure 1 presents the PRISMA flow diagram. A total of 1534 articles were identified through keyword and reference searches. After duplicate removal, 832 unique articles were subjected to title and abstract screening, resulting in the exclusion of 773. The full texts of the remaining 60 articles were then assessed against the study selection criteria, leading to the exclusion of 46 articles. The reasons for exclusion were as follows: no health-related outcome and nutritional biomarker reported (*n* = 23), children or adolescents with pre-existing chronic diseases besides undernutrition (*n* = 15), the inclusion of infants or adults only (*n* = 4), and exclusive tube feeding (*n* = 4). Therefore, 14 articles were included in the systematic review [41,42,43,44,45,46,47,48,49,50,51,52,53,54]. Among them, three reporting URTI incidences were included in the meta-analysis [42,44,48].

### 3.2. Characteristics of Study Participants

Table 1 summarizes the participant characteristics of the 14 included articles [41,42,43,44,45,46,47,48,49,50,51,52,53,54], presenting results from 13 unique studies (Vijayalakshmi et al. (2008a) [52] and Vijayalakshmi et al. (2008b) [53] reported results from the same intervention). These articles were published between 2000 and 2021, and about two-thirds (9 out of 14) were published after 2010 [44,45,46,47,48,49,50,51,53]. The studies recruited children and adolescents from 14 countries or regions, including Brazil [43], China [50], India [44,48,52,53], Mexico [43], Pakistan [41], Philippines [42,46], Portugal [43], Qatar [51], Spain [43], Taiwan [42], Turkey [47], US [45], Vietnam [49], and Korea [54]. Participants’ undernutrition status was determined using standard thresholds of anthropometric indicators, including weight-for-age, height-for-age, weight-for-height, and BMI [41,42,43,44,45,46,47,48,49,50,51,52,53,54]. Three study designs were adopted: RCT (*n* = 8) [42,43,44,45,48,50,51,52,53], pre-post study (*n* = 4) [41,46,49,54], and observational study (*n* = 1) [47]. The mean and median sample sizes are 231 and 142 participants, respectively, ranging between 20 and 842 participants [41,42,43,44,45,46,47,48,49,50,51,52,53,54]. Participants’ ages ranged from 1 to 14 years [41,42,43,44,45,46,47,48,49,50,51,52,53,54]. Boys averaged 56% in the samples, ranging between 46% and 100% [41,42,43,44,45,46,47,48,49,50,51,52,53,54].

### 3.3. Characteristics of the Included Studies

The characteristics of the included studies are summarized in Table 2. Among the eight RCTs [42,43,44,45,48,50,51,52,53], seven randomized participants to two arms—a treatment arm and a control arm [42,43,44,45,48,50,51], and the remaining intervention randomized participants to three arms—two treatment arms (one receiving ONS and the other receiving water-based micronutrient supplement) and a control arm [52,53]. Across trials, the treatment arms received ONS alone or in combination with other supplements (e.g., synbiotics) or therapies (e.g., growth hormone therapy, nutrition counseling). The control arms received nothing, therapy alone or ONS alone (when the corresponding treatment arm received ONS plus synbiotics) [42,43,44,45,48,50,51,52,53]. The four pre-post interventions measured and compared outcomes for all participants before and after ONS administration [41,46,49,54]. The observational study provided high-fiber ONS to all participants [47]. Nine studies were conducted in hospitals or clinics [41,44,45,46,47,48,50,51,54], three in schools or daycare centers [43,49,52,53], and the remaining one was administered on their team’s study site [42]. The intervention duration had a mean and median of 7 and 6 months, respectively, ranging from 0.5 to 18 months [41,42,43,44,45,46,47,48,49,50,51,52,53,54]. Eight of the 13 studies used PediaSure^®^ ONS product by Abbott Nutrition [41,42,43,44,45,46,47,49]; one used S-26 PE Gold^®^ ONS product by Wyeth Nutrition (Singapore) Pte. Limited [50]; one used Pediapowder^®^ ONS product by MDwell (Seoul, Republic of Korea) [54]; and the remaining three did not specify the ONS brand used [48,51,52,53]. The daily dosage of ONS varied substantially across interventions and was quantified using a lower bound, a fixed amount, or an intake frequency.

Two studies should be noted. Fisberg et al. (2002) compared ONS plus synbiotics (treatment arm) with ONS alone (control arm) [43]. Because our review focuses on the effectiveness of ONS and it is common for ONS to be added with synbiotics, we made inferences from this study by comparing the pre-treatment and post-treatment outcomes in both arms. Soliman et al. (2021) compared energy-dense (1.5 kcal/mL) ONS (treatment arm) with standard (1 kcal/mL) ONS (control arm) [51]. We made inferences from this study by comparing the relevant outcomes before and after consuming each ONS among both arms.

### 3.4. Intervention Effectiveness

Table 3 reports the intervention effectiveness among the included studies. Six studies exclusively reported acute disease and infection measures [43,44,46,47,48,50,53], five exclusively reported nutritional biomarkers [41,45,49,51,52,54], and the remaining one reported both [42]. Health-related outcomes include the number of sick days, degree of morbidity, number of acute illnesses, upper or lower respiratory tract infections, diarrhea, nausea, vomiting/regurgitation, abdominal distention, belching/burping, flatulence, constipation, and stool frequency/consistency [42,43,44,46,47,48,50,53]. Nutritional biomarkers (and other biochemical indices) include serum sodium, chloride, iron, calcium, phosphorus, zinc, potassium, blood urea nitrogen (BUN), creatinine, total protein, C-reactive protein, ferritin, glucose, insulin, pre-albumin, albumin, triglyceride, alkaline phosphatase, uric acid, total bilirubin, high-density lipoprotein (HDL) cholesterol, lipids, white blood count (WBC), hemoglobin, platelet, hematocrit, neutrophil, lymphocyte, monocyte, and eosinophil, growth hormones, transferrin, acylated ghrelin, total iron binding capacity, IGF-1, IGFBP-3, and α-1-acid glycoprotein (AGP) [41,42,45,49,50,52,54].

Two key findings emerged from the estimated intervention effects on health-related outcomes and nutritional biomarkers. First, six of the eight studies measuring health-related outcomes reported statistically significant improvement in some but not all outcomes. Specifically, Fisberg et al. (2002) found that the number of sick days per month decreased over four months among both ONS feeding groups with and without synbiotics added [43]. Alarcon et al. (2003) and Ghosh et al. (2018) found that the ONS intervention reduced URTI incidence [42,44]. Huynh et al. (2015) found that the number of sick days decreased following the ONS intervention [46]. Kansu et al. (2018) found that the ONS intervention was associated with reduced vomiting, nausea, abdominal distension, and improved stool frequency [47]. Vijayalakshmi et al. (2008) showed that compared to the control group, supplementation with ONS mixed in milk and ONS prepared using water both resulted in significant improvement in the degree of morbidity [53]. On the other hand, Alarcon et al. (2003) identified no change in gastrointestinal symptoms (e.g., diarrhea, constipation) [42]. Sheng et al. (2014) and Khadilkar et al. (2021) found no effect of the ONS intervention on the incidence of acute illnesses [48,50].

Second, five of the seven studies evaluating nutritional biomarkers and other relevant biochemical indices reported statistically significant improvement in some but not all measures. Specifically, Akram et al. (2000) found the ONS intervention associated with improved serum sodium, potassium, BUN, creatinine, calcium, phosphorus, total protein, cholesterol, and triglyceride [41]. Vijayalakshmi et al. (2008) found that the mean blood hemoglobin levels of children aged 7–12 years significantly increased after receiving a milk-based ONS when compared to the control group [52]. Additionally, they observed a significant improvement in mean blood hemoglobin levels for children aged 7 and 10 years after using ONS prepared with water [52]. However, there was no significant improvement in mean blood hemoglobin levels for children aged 8, 9, 11, and 12 years when using the water-based ONS [52]. Pham et al. (2020) found that the ONS intervention improved blood hemoglobin, albumin, and zinc concentrations and reduced albumin and zinc deficiency [49]. Soliman et al. (2021) found that ONS consumption improved IGF-1 among undernourished children [51]. Shim et al. (2020) reported an increase in blood urea nitrogen concentration following ONS consumption over a 6-month intervention period [54]. On the other hand, Alarcon et al. (2003) found no improvement in serum albumin, iron, ferritin, and zinc [42]. Han et al. (2011) reported no improvement in IGF-1, IGFBP-3, transferrin, pre-albumin, ghrelin, and lipid concentrations [45].

### 3.5. Meta-Analysis

**Figure 2:**
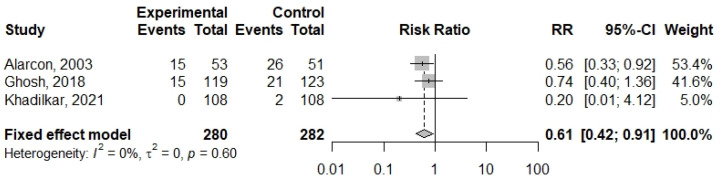
Forest plot of the meta-analysis on the incidence of upper respiratory tract infection [42,44,48].

A fixed-effect meta-analysis was performed on the outcome of URTI incidence, which was reported by Alarcon et al. (2003), Ghosh et al. (2018), and Khadilkar et al. (2021) [42,44,48]. The ONS intervention was estimated to reduce URTI incidence by 39% (pooled risk ratio = 0.61; 95% confidence interval [CI] = 0.42, 0.91; *p*-value = 0.01; *I*^2^ index = 0.00%) in undernourished children and adolescents. Figure 2 shows the forest plot of the meta-analysis estimate. No publication bias test was feasible due to the small sample size (*n* = 3).

### 3.6. Study Quality Assessment

We assessed the evidence/quality of the studies included in the review using the GRADE framework [40]. As shown in Table 1, five studies were rated “high”, two “moderate”, and the remaining seven “low.” The primary reason for a “low” rating concerns a non-randomized study design (observational or pre-post study). Other reasons include the risk of bias (i.e., potential confounders correlated with the treatment and outcomes) and imprecision (e.g., lacking quantitative estimates of the treatment effects concerning specific outcome measures).

## 4. Discussion

This study systematically reviewed the scientific evidence concerning the effects of ONS on health-related outcomes and nutritional biomarkers in children and adolescents with undernutrition. A comprehensive keyword and reference search in seven electronic bibliographic databases identified 14 peer-reviewed articles reporting results from 13 unique studies, including eight RCTs, four pre-post studies, and one observational study [41,42,43,44,45,46,47,48,49,50,51,52,53,54].

### 4.1. Health-Related Outcomes

Six of the eight studies examining health-related outcomes reported statistically significant improvement in some but not all outcomes [42,43,44,46,47,48,50,53]. A meta-analysis of three studies [42,44,48] found that ONS interventions reduced URTI incidence in undernourished children by 39% (95% CI, 0.42–0.91). This finding underscores the importance of adequate nutrition in supporting immune function.

Adequate nutrition is essential in supporting immune response [55,56]. Poor nutrition may impair host defenses by affecting both the innate and adaptive immune systems [8,57]. Undernutrition has also been linked to intestinal dysbiosis [58,59,60], an undesirable alteration of the microbiota resulting in an imbalance between beneficial and harmful bacteria. It can impact the immune system since intestinal microbiota is essential in regulating systemic immunity and gut barrier function [59,61].

ONS are energy- and nutrient-dense formulations, containing both macro- and micronutrients and often added with prebiotics or probiotics [62]. To our knowledge, this meta-analysis is the first to demonstrate that ONS usage decreased the risk of upper respiratory tract infections in undernourished children. Repeated infections can aggravate undernutrition due to appetite loss, nutrient malabsorption, increased demand for nutrients, and diversion of nutrients for immune response [6,13,63]. Our finding suggests that ONS can improve the immunity of undernourished children, thus helping them escape the “vicious cycle” of undernutrition infection [6,7,8]. This current result complements findings from an earlier systematic review, which showed the effectiveness of ONS intervention in enhancing better growth outcomes for children suffering from undernutrition compared to control treatments [29].

### 4.2. Nutritional Biomarkers

Nutritional biomarkers can generally be interpreted as the biological consequence of dietary intake [64]. As the use of ONS should help restore nutrient deficiencies, it is expected that nutritional biomarkers of undernourished children will improve or regain normality. However, this is not universally seen based on the results of our systematic review.

It is recognized that a range of factors, including genetic variability, lifestyle, and analytical methodology, can influence biomarker measures of dietary intake [65,66]. Furthermore, evidence suggests that specific biomarkers, such as total white blood cell and lymphocyte counts, are not affected by undernutrition [67]. It is thus plausible that nutritional repletion may not always result in a change in biochemical parameters. The inconsistent findings observed could also be attributed to differences in the severity of nutritional deficiency, amount of ONS provided, and duration of nutrition intervention. These discrepancies highlight the need for standardized protocols and more rigorous studies to fully understand the impact of ONS on nutritional biomarkers. Nevertheless, robust evidence, including data from this review, has shown that ONS usage among undernourished children results in clinically meaningful endpoints, such as gains in weight and height and a reduced risk of infection. Hence, despite inconsistent findings on nutritional biomarkers due to various factors discussed above, the positive outcomes observed in growth and immunity can be indicative of an overall improvement of nutrient status following ONS supplementation.

A primary strength of this study is that it represents the first systematic review to assess the effectiveness of ONS on health-related outcomes and nutritional biomarkers in children and adolescents with undernutrition. We aimed to perform a rigorous review by adhering to established guidelines for such analyses, which were in line with the Cochrane Collaboration’s recommendations for intervention studies [32]. However, it is important to acknowledge several limitations related to both the review and the studies included. The literature examining relevant outcomes for undernourished children and adolescents undertaking an ONS intervention remains small and heterogeneous. Participants resided in 14 countries or regions with a wide age range. The interventions differed substantially in design, duration, ONS product used, and daily dosage administered. Pertinent measures were extensive and diverse, preventing meta-analysis and robust conclusions on the treatment effect on a specific outcome. Some studies failed to report the quantitative estimates of the treatment effect for the full range of outcomes assessed, and select reporting could be prone to publication bias. The review scope was limited to energy-dense, oral ONS products (≥ 1 kcal/mL); other nutrient-rich products, such as fortified milk and functional food, and enteral tube feeding of ONS were not considered. We excluded undernourished children and adolescents hospitalized or with pre-existing chronic conditions, as they may need medical treatment and health care besides nutrition intervention to restore health and normal growth. The meta-analysis estimate was based on only three studies and may lack generalizability. Moreover, their study samples were recruited from diverse geographical regions (Philippines and Taiwan vs. India), and interventions were performed in different settings (daycare center vs. hospital/clinic). Finally, children and adolescents participating in the studies included in the review could differ in their malnutrition (e.g., mild, moderate, or severe undernutrition) and health status, leading to differential responses to the ONS treatment. 

## 5. Conclusions

This systematic review and meta-analysis demonstrate the potential benefit of ONS for undernourished children and adolescents in terms of improving certain health-related outcomes and nutritional biomarkers. Notably, the use of ONS was found to reduce the incidence of URTI by 39%, emphasizing its role in enhancing immune function among children with or at risk of undernutrition. This may have a significant impact on optimizing the growth and development of these children, considering URTI is a very common infection during childhood [68]. While some improvements in nutritional biomarkers were observed, the results were inconsistent across all studies, suggesting variability due to genetic differences, lifestyle, and methodological variations.

Overall, our findings underscore the importance of incorporating ONS into nutritional interventions aimed at this vulnerable population to help improve their overall health and nutritional status. Future research should focus on standardizing ONS formulations and intervention protocols to further elucidate their effects and optimize outcomes. Exploring the long-term impact of ONS intake on growth, cognitive development, and overall health in this population would provide valuable insights for policymakers and healthcare providers.

## Figures and Tables

**Table 1:** Characteristics of the studies included in the review.

Study ID	Author, Year	Country	Undernutrition Status	Study Design	Sample Size	Age Range	Mean Age ± SD	% Boys	Grade Quality
1	Akram, 2000 [41]	Pakistan	weight-for-age < −3 SD	Pre-post	30	1–5 y	22.0 ± 8.6 m	50	Low
2	Fisberg, 2002 [43]	Brazil, Mexico, Portugal, Spain	−1 SD < weight-for-height < −3 SD	RCT	626	1–6 y	Treatment arm: 3.5 ± 2.0 y Control arm: 3.5 ± 2.3 y	53	Moderate
3	Alarcon, 2003 [42]	Philippines, Taiwan	weight-for-height < 25th percentile	RCT	104	3–5 y	48.5 m	58	High
4	Vijayalakshmi, 2008a [52]	India	N/A	RCT	842	7–12 y	N/A	50	Low
5	Vijayalakshmi, 2008b [53]	India	N/A	RCT	842	7–12 y	N/A	50	Low
6	Han, 2011 [45]	USA	height < −2 SD, bone age < 10 y and delayed by > 1 y	RCT	20	7–11 y	9.3 ± 1.3 y	100	High
7	Sheng, 2014 [50]	China	weight-for-height < 25th percentile	RCT	142	3–5 y	Treatment arm: 3.8 ± 0.7 y Control arm: 3.6 ± 0.7 y	46.5	High
8	Huynh, 2015 [46]	Philippines	5th percentile < weight-for-height < 25th percentile	Pre-post	199	3–4 y	41.2 ± 3.6 m	49.7	Low
9	Ghosh, 2018 [44]	India	−2 < weight-for-age *z*-scores < −1	RCT	255	2–6 y	44.0 ± 14.3 m	62.7	High
10	Kansu, 2018 [47]	Turkey	weight-for-age < −2 SD, height-for-age < −2 SD	Observational	345	1–10 y	4.8 ± 2.7 y	48.4	Low
11	Pham, 2020 [49]	Vietnam	height-for-age *z*-scores < −2, weight-for-height *z*-scores < −1	Pre-post	106	2–4 y	N/A	47.2	Low
12	Shim, 2020 [54]	Korea	weight-for-height *z*-score < −2	Pre-post	82	1–3 y	21.6 ± 7.3 m	61	Low
13	Khadilkar, 2021 [48]	India	−2 < weight-for-age *z*-scores < −1	RCT	216	4–6 y	Treatment group: 4.5 ± 0.6 y Control group: 4.6 ± 0.6 y	46.3	High
14	Soliman, 2021 [51]	Qatar	height-for-age < −2, 2 SD < standardized BMI < −1 SD	RCT	34	5–14 y	10.2 y	N/A	Moderate

Notes: RCT—randomized controlled trial; BMI—body mass index; N/A—not available.

**Table 2:** Intervention characteristics.

Study ID	Author, Year	Intervention Type	Intervention Setting	Intervention Duration	Arm-Specific Sample Size
1	Akram, 2000 [41]	Pre: no ONS Post: ONS	Hospital/clinics	0.5 m	Single arm: 30
2	Fisberg, 2002 [43]	Treatment arm: ONS + synbiotics Control arm: ONS	Daycare center	4 m	Treatment arm: 310 Control arm: 316
3	Alarcon, 2003 [42]	Treatment arm: NC + ONS Control arm: NC	Study site	3 m	Treatment arm: 53 Control arm: 51
4	Vijayalakshmi, 2008a [52]	Treatment arm 1: ONS (milk-based micronutrient supplement) Treatment arm 2: water-based micronutrient supplement Control group: no ONS	School	12 m	Treatment arm 1: 270 Treatment arm 2: 291 Control arm: 281
5	Vijayalakshmi, 2008b [53]	Treatment arm 1: ONS (milk-based micronutrient supplement) Treatment arm 2: water-based micronutrient supplement Control group: no ONS	School	12 m	Treatment arm 1: 270 Treatment arm 2: 291 Control arm: 281
6	Han, 2011 [45]	Treatment arm: 6 m ONS + 12 m daily G.H. therapy Control group: 6 m observation + 12 m daily GH therapy	Hospital/clinics	18 m	Treatment arm: 10 Control arm: 10
7	Sheng, 2014 [50]	Treatment arm: NC + ONS Control arm: NC	Hospital/clinics	4 m	Treatment arm: 75 Control arm: 67
8	Huynh, 2015 [46]	Pre: no ONS Post: ONS	Hospital/clinics	12 m	Single arm: 199
9	Ghosh, 2018 [44]	Treatment arm: NC + ONS Control arm: NC	Hospital/clinics	3 m	Treatment arm: 127 Control arm: 128
10	Kansu, 2018 [47]	All subjects: ONS (high-fiber)	Hospital/clinics	6 m	Single arm: 345 Baseline: 345 1st follow-up visit (2–3 m): 126 2nd follow-up visit (4–6 m): 138
11	Pham, 2020 [49]	Pre: no ONS Post: ONS	School	6 m	Single arm: 106
12	Shim, 2020 [54]	Good consumption group: consumed ≥ 60% of the recommended dose of the formula) Poor consumption group: consumed < 60% of the recommended dose of the formula)	Hospital/clinics	6 m	Good consumption group: 38 Poor consumption group: 44
13	Khadilkar, 2021 [48]	Treatment arm: NC + ONS Control arm: NC	Hospital/clinics	3 m	Treatment arm: 108 Control arm: 108
14	Soliman, 2021 [51]	Treatment arm: ONS (energy-dense) Control arm: ONS (standard)	Hospital/clinics	12 m	Treatment arm: 22 Control arm: 12

Notes: GH—growth hormone; ONS—oral nutritional supplement; NC—nutrition counseling.

**Table 3:** Intervention effectiveness.

Study ID	Author, Year	ONS Product Characteristics	ONS Daily Dose	Nutritional Biomarkers and Other Biochemical Indices	Health-Related Outcomes	Intervention Effectiveness
1	Akram, 2000 [41]	PediaSure (Abbott): 1 kcal/mL, 3 g of protein/kcal, 49.8 g of total fat/L, and 109.7 g of carbohydrate/L	The quantity of PediaSure administered varied by child according to demand and tolerance. The minimum energy administered for each child was at least 50% of the total requirement based on weight-for-age.	Serum electrolyte, sodium, potassium, chloride, BUN creatinine, calcium, phosphorus, total protein, fasting/random glucose, albumin, cholesterol, triglyceride, alkaline phosphatase, uric acid, total bilirubin, HDL, WBC, Hb, platelet, hematocrit, neutrophil, lymphocyte, monocyte, and eosinophil.		Sodium (mEq/L) ↑ (Pre: 136.4 ± 3.7; Post: 139.2 ± 3.1; *p* < 0.001) Potassium (mEq/L) ↑ (Pre: 4.4 ± 0.9; Post: 5.4 ± 0.6; *p* < 0.001) BUN (mg %) ↑ (Pre: 6.6 ± 2.7; Post: 12 ± 3.7; *p* < 0.001) Creatinine (mg %) ↓ (Pre: 0.4 ± 0.1; Post: 0.3 ± 0.1; *p* < 0.001) Calcium (mg %) ↑ (Pre: 9.4 ± 0.8; Post: 9.8 ± 0.7; *p* < 0.05) Phosphorus (mg %) ↑ (Pre: 4.1 ± 1.1; Post: 5.8 ± 1.1; *p* < 0.001) Total protein (g %) ↑ (Pre: 6.8 ± 0.9; Post: 7.3 ± 0.8; *p* < 0.05) Cholesterol (mg %) ↑ (Pre: 111 ± 36; Post: 136 ± 38; *p* < 0.05) Triglyceride (mg %) ↓ (Pre: 200 ± 98; Post: 188 ± 90; *p* < 0.05) There was no statistically significant change for all other outcome measures before and after the intervention.
2	Fisberg, 2002 [43]	PediaSure (Abbott) + synbiotics: FOS (0.5g/L after reconstitution) and probiotic bacteria bifidobacterium and acidophilus, each at the level of 3 × 10^7^ CFU/g.	Subjects were expected to consume between 375 and 750 mL/d of their assigned supplement for at least 80% of the 4 m study period.		Sick days, diarrhea, upper and lower respiratory tract infection, stool frequency and consistency.	Within-arm comparisons: Treatment arm: Number of sick days per month: ↓ (*p* < 0.001) Constipation days: ↓ (*p* < 0.05) Control arm: Number of sick days per month: ↓ (*p* < 0.001) Between-arm comparisons (treatment arm vs. control arm): Number of sick days: no significant change Incidence of illness: no significant change Number of sick episodes: no significant change Number of subjects who experienced diarrhea: no significant change Days of diarrhea: no significant change Episodes of diarrhea that required antibiotic treatment: no significant change Mean stool consistency or frequency: no significant change Number of days of constipation: lower in the experimental group than in the control group (*p* < 0.05) Subgroup comparisons: Number of sick days/m decreased over 4 m more pronounced in 1–2 y children (*p* < 0.001) Treatment arm experienced significantly reduced sick days in 3–5 y children
3	Alarcon, 2003 [42]	PediaSure (Abbott): lactose-free, providing 1.0 kcal/mL (12% protein, 43.8% carbohydrate, and 44.8% fat).	Subjects in the treatment arm were to consume 40 mL/kg/d of the supplement in addition to their regular diet and not to consume any similar products during the study.	Serum albumin, iron, ferritin, and zinc.	Gastrointestinal symptoms include diarrhea, constipation, nausea, vomiting or regurgitation, abdominal distention, belching or burping, and flatulence.	Between-arm comparisons (treatment arm vs. control arm): Change in gastrointestinal symptom scores: no significant change Rate of URTI: 28% vs. 51% (*p* < 0.05) Serum iron: 29% vs. 7% (*p* > 0.05) Serum zinc: 11% vs. 7% (*p* > 0.05)
4	Vijayalakshmi, 2008a [52]	Treatment arm 1: 66 g of micronutrient supplement mixed in 300 mL of milk. Treatment arm 2: 66 g of micronutrient supplement mixed in 300 mL of water.	Twice a day for 6 d in a week for a duration of 12 m except Sundays and long holidays.	Hb		Within-arm comparisons: Treatment arm 1: Mean blood Hb levels (g/dL): ↑ 1.12 for 7 years old ↑ 1.40 for 8 years old ↑ 1.44 for 9 years old ↑ 1.40 for 10 years old ↑ 1.42 for 11 years old ↑ 1.46 for 12 years old Treatment arm 2: Mean blood Hb levels (g/dL): ↑ 1.22 for 7 years old ↑ 0.96 for 8 years old ↑ 0.90 for 9 years old ↑ 0.94 for 10 years old ↑ 0.94 for 11 years old ↑ 0.98 for 12 years old Control arm: Mean blood Hb levels (g/dL): ↑ 0.74 for 7 years old ↑ 0.80 for 8 years old ↑ 0.76 for 9 years old ↑ 0.38 for 10 years old ↑ 0.88 for 11 years old ↑ 0.14 for 12 years old Between-arm comparisons (treatment arm vs. control arm) Mean blood Hb levels (g/dL): Treatment arm 1 vs. control arm: *p* < 0.05 for 7 and 9 years old; *p* < 0.01 for 8, 10, 11, and 12 years old Treatment arm 2 vs. control arm: *p* < 0.05 for 7 and 10 years old; not significant for 8, 9, 11, and 12 years old
5	Vijayalakshmi, 2008b [53]	Treatment arm 1: 66 g of micronutrient supplement mixed in 300 mL of milk. Treatment arm 2: 66 g of micronutrient supplement mixed in 300 mL of water.	Twice a day for 6 d in a week for a duration of 12 m except Sundays and long holidays.		Morbidity patterns	Within-arm comparisons: Treatment arm 1: Free from morbidity: ↑ 36.3% (Baseline: 10.37%; Mid: 33.33%; Post: 46.67%) First-degree morbidity: ↑ 2.59% (Baseline: 38.89%; Mid: 40.00%; Post: 41.48%) Second-degree morbidity: ↓ 32.96% (Baseline: 44.81%; Mid: 25.93%; Post: 11.85%) Third-degree morbidity: ↓ 5.93% (Baseline: 5.93%; Mid: 0.74%; Post: 0%) Treatment arm 2: Free from morbidity: ↑ 26.08% (Baseline: 7.22%; Mid: 25.43%; Post: 33.30%) First-degree morbidity: ↑ 18.9% (Baseline: 28.18%; Mid: 41.24%; Post: 47.08%) Second-degree morbidity: ↓ 34.02% (Baseline: 53.61%; Mid: 32.30%; Post: 19.59%) Third-degree morbidity: ↓ 10.99% (Baseline: 10.99%; Mid: 1.03%; Post: 0%) Control arm: Free from morbidity: ↓ 3.92 (Baseline: 16.01%; Mid: 14.59%; Post: 12.09%) First-degree morbidity: ↑ 7.12% (Baseline: 32.03%; Mid: 46.26%; Post: 39.15%) Second-degree morbidity: ↑ 3.21% (Baseline: 42.70%; Mid: 37.01%; Post: 45.91%) Third-degree morbidity: ↓ 6.4% (Baseline: 9.25%; Mid: 2.14%; Post: 2.85%)
6	Han, 2011 [45]	PediaSure (Abbott): 237 kcal and 7 g protein/8-oz can. G.H. therapy: dosed at 0.3 mg/kg/w administered subcutaneously once daily (Nutropin AQ, Genentech).	The children were prescribed, on average, 10.4 ± 2.5 oz ONS daily (i.e., energy 13.4 kcal/kg/d and protein 0.4 g/kg/d), with subsequent adjustments based on individual weight and energy needs (6 m: 16.8 ± 2.0 oz/d; 12 m: 20.0 ± 1.9 oz/d; 18 m: 22.0 ± 2.1 oz/d). GH therapy: GH was dosed at 0.3 mg/kg/w and administered subcutaneously once daily.	Growth hormones, pre-albumin, lipids, transferrin, IGF-1, IGFBP-3, pre-albumin, acylated ghrelin, fasting insulin, and glucose.		Within-arm comparisons: IGF-1 concentration: ↑ at 12 m and 18 m within both arms (*p* < 0.05) Transferrin: ↑ at 12 m within control arm (*p* < 0.01) HOMA-IR: ↑ at 12 m within control arm (*p* < 0.05) IGFBP-3: no significant change Pre-albumin: no significant change Ghrelin: no significant change Lipid concentrations: no significant change Between-arm comparisons (treatment arm vs. control arm): IGF-1 concentration: no significant change Transferrin: no significant change HOMA-IR: no significant change IGFBP-3: no significant change Pre-albumin: no significant change Ghrelin: no significant change Lipid concentrations: no significant change
7	Sheng, 2014 [50]	The ONS was a milk-based powder (S-26 PE GOLD, Wyeth Nutrition, Singapore). The ONS provided 200 kcal/serving (14% protein, 54% carbohydrate, and 32%. fat) and micronutrients. Amount per serving (230 mL): energy 200 kcal, protein 7 g, fat 7 g, arachidonic acid 5.2 mg, docosahexaenoic acid 3.6 mg, carbohydrates 27 g, nucleotides 5.2 mg, taurine 9.4 mg, l-carnitine 3.4 mg, lutein 40 mcg, vitamin A 200 mcg, carotenes 42 mcg, vitamin D 3.3 mcg, vitamin E 2.2 mg, vitamin K 13 mcg, vitamin B_1_ 0.26 mg, vitamin B_2_ 320 mcg, vitamin B_6_ 250 mcg, vitamin B_12_ 0.5 mcg, niacin 1388 mcg, folic acid 19 mcg, pantothenic acid 1000 mcg, biotin 4.9 mcg, vitamin C 24 mg, choline 60 mg, inositol 15 mg, calcium 260 mg, phosphorus 170 mg, magnesium 28 mg, iron 3.8 mg, zinc 2.4 mg, manganese 225 mcg, copper 175 mcg, iodine 19 mcg, sodium 118 mg, potassium 475 mg, and chloride 275 mg.	At least two 230 mL servings per day.		Diarrhea, and upper and lower respiratory tract infections.	Between-arm comparisons (treatment arm vs. control arm): Incidence of common illnesses: no significant change
8	Huynh, 2015 [46]	PediaSure (Abbott) ONS (450 mL): energy 450 kcal, protein 13.5 g, vitamin A 270 mcg, vitamin C 45 mg, vitamin B_1_ 1.4 mg, vitamin B_2_ 0.9 mg, niacin 6.8 mg, vitamin B_6_ 1.2 mg, vitamin B_12_ 1.4 mcg, vitamin D 9.0 mcg, vitamin E 7.2 mg, folate 112.5 mcg, calcium 432 mg, iron 6.3 mg, iodine 43.7 mcg, magnesium 89.1 mg, phosphorus 373.5 mg, zinc 3.0 mg, selenium 14.4 mcg, manganese 0.7 mg, lactobacillus acidophilus 3.9 × 10^7^ CFU, bifidobacterium 2.45 × 10^6^ CFU, and FOS 1.98 g.	Two servings of ONS per day for 48 w, the ONS provided 450 kcal, 13.5 g of high-quality protein, 17.7 g of easily-digested fat and 59.4 g of carbohydrate, and 28 minerals and vitamins (450 mL in total).		Sick days and number of acute illnesses.	Number of sick days significantly decreased from 16 w onwards (*p* < 0.0001)
9	Ghosh, 2018 [44]	PediaSure (Abbott), ONS per serving (45.5 g): energy 213 kcal, protein 6.4 g, fat 10.6 g, carbohydrate 22.8 g, vitamin A 138.8 mcg, vitamin C 20.0 mg, vitamin B_1_ 0.41 mg, vitamin B_2_ 0.46 mg, niacin 3.19 mg, vitamin B_6_ 0.46 mg, vitamin B_12_ 0.68 mg, pantothenic acid 1.41 mg, biotin 7.28 mcg, choline 53.7 mg, vitamin D_2_ 1.43 mcg, vitamin E 5.01 mg, vitamin K 7.96 mcg, folic acid 45.5 mcg, calcium 175.6 mg, iron 2.5 mg, iodine 17.3 mcg, magnesium 89.1 mg, phosphorus 109.2 mg, zinc 1.59 mg, selenium 5.60 mcg, and manganese 0.45 mg.	Children 24–48 m: at least 224 mL ONS; Children 48–72 m: 448 mL of ONS.		Sick days from URTI and number of acute URTI episodes.	Recurrent URTI incidence in the control arm was 2.01 times higher than in the treatment arm. The incidence rate of URTI was 13.0% and 17.0% for the intervention and the control groups, respectively.
10	Kansu, 2018 [47]	PediaSure Fiber version (Abbott): protein (11.2%), carbohydrate (43.6%), fat (44.7%) and dietary fiber and short-chain FOS (0.5%). Fortini 1.0 Multi Fibre (Nutricia): carbohydrates (10.0%), fat (47%), and dietary fiber (3.0%).	High-fiber regimens were used as ONS based on an average of 2 packages/d in the majority of patients that provided daily calorie intakes of at least 40 kcal/kg, daily fiber intake of at least 300 mg/kg and daily water intake of at least 25 mL/kg in half of the patients.		Comorbidity, gastrointestinal symptoms, and defecation habits.	Percentage of gastrointestinal symptom-free patients: (a) Vomiting: ↑ (Baseline: 85.7%; 4–6 m: 92.8%; *p* < 0.01); (b) Nausea: ↑ (Baseline: 82.5%; 4–6 m: 91.3%; *p* < 0.01); (c) Abdominal distension: ↑ (Baseline: 90.5%; 4–6 m: 93.5%; *p* < 0.001)
11	Pham, 2020 [49]	PediaSure (Abbott): Each cup consisted of 190 mL of cooled boiled water (<37 °C) mixed with 5 tablespoons brushed-across PediaSure powder (equivalent to 49 g of flour) and stirred 225 mL (1 mL is equivalent to 1 kcal).	Each child was given 2 glasses of PediaSure per d continuously for 6 m.	Hb, albumin, zinc, CRP, and AGP.		Hb (g/L): ↑ (Pre: 112.05 ± 8.6; Post: 117.9 ± 7.6; *p* < 0.05) Albumin (g/L): ↑ (Pre: 32.3 ± 3.0; Post: 41.1 ± 4.1; *p* < 0.05) Zinc concentrations (µmol/L): ↑ (Pre: 10.1 ± 1.1; Post: 11.0 ± 0.96; *p* < 0.05) The anemia rate: ↓ (Pre: 29.2%; Post: 10.4%; *p* < 0.05). Albumin deficiency: ↓ (Pre: 82.1%; Post: 20.8%; *p* < 0.05) Zinc deficiency: ↓ (Pre: 66.0%; Post: 29.2%; *p* < 0.05) Subgroup comparisons: Girls had more significant improvement than boys in Hb, albumin, and zinc. The 24-m to 36-m age groups had more significant improvement in biochemical indices.
12	Shim, 2020 [54]	Pediapowder (MDwell, Seoul, Korea) (400 mL): calorie 400 kcal, protein 12 g, lipid 14 g, carbohydrate 56 g, vitamin A 250 μg, vitamin D 4 μg, vitamin E 10 mg, vitamin K 26 μg, vitamin C 40 mg, vitamin B_1_ 1 mg, vitamin B_2_ 0.84 mg, vitamin B_6_ 1 mg, vitamin B_12_ 2.48 μg, Niacin 3.32 mg, Biotin 7.2 μg, folic acid 100 μg, pantothenic acid 4.16 mg, inositol 33.2 mg, calcium 422 mg, phosphorus 340 mg, potassium 516 mg, iron 4.48 mg, magnesium 44 mg, sodium 120 mg, zinc 4.4 mg, manganese 0.68 mg, and copper 0.44 mg.	Children were instructed to consume 2 sachets of the formula, Pediapowder^®^ (MDwell, Seoul, Korea), mixed with 400 mL of water per day.	Blood urea nitrogen concentration, serum concentration of calcium, iron, and total iron binding capacity, ferritin, uric acid, total bilirubin, hemoglobin, albumin, and creatinine.		Within-arm comparisons: Good consumption group Serum concentration of calcium, iron, and total iron binding capacity: ↑ (*p* < 0.05) Serum concentration of ferritin: ↓ (*p* < 0.05) Serum levels of uric acid, total bilirubin, and ferritin: ↓ (*p* < 0.05) Poor consumption group Serum concentration of calcium, iron, and total iron binding capacity: ↑ (*p* < 0.05) Serum concentration of ferritin: ↓ (*p* < 0.001) Levels of hemoglobin, albumin, creatinine, iron, and total iron binding capacity: ↑ (*p* < 0.05) Between-arm comparisons: Blood urea nitrogen concentration: ↑ (*p* = 0.001) There was no statistically significant difference for the other laboratory parameters between the two groups (*p* > 0.05)
13	Khadilkar, 2021 [48]	ONS per serving (45 g): energy 201 kcal, protein 8 g, carbohydrates 27 g, and total fat 7 g.	45 g of ONS daily.		Incidences of fever, vomiting, cold, diarrhea, cough, URTI, and rashes.	Between-arm comparisons (treatment arm vs. control arm): Fever: 5 (4.6%) vs. 8 (7.4%) Vomiting: 4 (3.7%) vs. 0 Cold: 3 (2.7%) vs. 2 (1.8%) Diarrhea: 1 (0.9%) vs. 0 Cough: 1 (0.9%) vs. 0 URTI: 0 vs. 2 (1.8%) Rashes: 0 vs. 1 (0.9%)
14	Soliman, 2021 [51]	Energy-dense ONS per 200 mL bottle: energy 300 kcal, energy density 1.5 kcal/mL, protein 6.8 g, carbohydrates 37.6 g, and fat 13.6 g. Standard ONS per 200 mL bottle: energy 200 kcal, energy density 1 kcal/mL, protein 4.8 g, carbohydrates 23.6 g, and fat 9 g.	Treatment arm (energy-dense ONS): 1.5 kcal/mL. Control arm (standard ONS): 1 kcal/ml.	IGF-1		Within-arm comparisons: Treatment arm Change in IGF1-SDS: 1.5 ± 0.6, *p* < 0.05 Control arm Change in IGF1-SDS: 0.35 ± 0.16, *p* < 0.05 Between-arm comparisons (treatment arm vs. control arm): Change in IGF1-SDS: ↑ (1.5 ± 0.6 vs. 0.35 ± 0.16; *p* = 0.02)

Notes: HDL—high-density lipoprotein; WBC—white blood cell; BUN—blood urea nitrogen; IGF-1—insulin growth factor 1; ONS—oral nutritional supplement; CRP—C-reactive protein; AGP—Alpha-1 glycoprotein; Hb—hemoglobin; URTI—upper respiratory tract infection; and GH—growth hormone. ↑ denotes a statistically significant increase at *p* < 0.05; ↓ denotes a statistically significant decrease at *p* < 0.05.

## Data Availability

The data used in this publication are owned by Abbott Nutrition. Data access requests can be sent to dieu.huynh@abbott.com for evaluation.

## Appendix A. Search Algorithm

**Table:**

Search Platform	Databases	Search Algorithm
EBSCO	PubMed/MEDLINE Academic Search Complete Academic Search Premier CINAHL Global Health	( AB (“oral nutritional supplement*” or “oral nutrition supplement*” or “young child formula*” or PediaSure or Sustagen or NUTREN or “Boost Kid Essentials” or Kindercal or “Bright Beginnings” or “Peptamen Junior” or “S-26 PE Gold” or “Junior Horlicks” or Bournvita or Complan or Fortisip or Fortini or “Protinex Junior” or “Nutrilite Kids” or Dinoshake or “Nutraherbs Kids” or “Amul Pro” or Wyeth or “NG Solutions” or “Mead Johnson” or Nestle or Nutricia or ((nutrition or nutritional or nutrient or nutrients) N10 (“oral supplement*” or “dietary supplement*” or “diet supplement*”)) or “supplementary oral nutrition” or “supplemented oral nutrition” or “supplementary enteral nutrition” or “supplemented enteral nutrition” or “supplemental enteral nutrition” or ((oral or orally) and (“enteral formula*” or “enteric formula*” or “polymeric formula*” or “polymeric diet*”)) or “oral nutritional intervention*” or “sip feed*” or “nutrition drink” or “nutrition drinks” or “nutritional drink*” or “milk drink” or “milk drinks” or “fortified milk*” or “enriched milk*” or “fortified beverage*” or “liquid nutrition* supplement*” or “oral enteral nutrition*” or “oral enteric nutrition*” or “oral enteral feeding*” or “protein energy supplement*” or “protein-energy supplement*” or “protein and energy supplement*” or “protein calorie supplement*” or “protein-calorie supplement*” or “protein and calorie supplement*” or “oral nutrition*” or ((oral or orally) N10 nutrition* N10 supplement*) or ((oral or orally) and (“enteral nutrition*” or “enteral supplement*” or “enteral feeding*”) NOT (“tube feeding*” ))) OR TI (“oral nutritional supplement*” or “oral nutrition supplement*” or “young child formula*” or PediaSure or Sustagen or NUTREN or “Boost Kid Essentials” or Kindercal or “Bright Beginnings” or “Peptamen Junior” or “S-26 PE Gold” or “Junior Horlicks” or Bournvita or Complan or Fortisip or Fortini or “Protinex Junior” or “Nutrilite Kids” or Dinoshake or “Nutraherbs Kids” or “Amul Pro” or Wyeth or “NG Solutions” or “Mead Johnson” or Nestle or Nutricia or ((nutrition or nutritional or nutrient or nutrients) N10 (“oral supplement*” or “dietary supplement*” or “diet supplement*”)) or “supplementary oral nutrition” or “supplemented oral nutrition” or “supplementary enteral nutrition” or “supplemented enteral nutrition” or “supplemental enteral nutrition” or ((oral or orally) and (“enteral formula*” or “enteric formula*” or “polymeric formula*” or “polymeric diet*”)) or “oral nutritional intervention*” or “sip feed*” or “nutrition drink” or “nutrition drinks” or “nutritional drink*” or “milk drink” or “milk drinks” or “fortified milk*” or “enriched milk*” or “fortified beverage*” or “liquid nutrition* supplement*” or “oral enteral nutrition*” or “oral enteric nutrition*” or “oral enteral feeding*” or “protein energy supplement*” or “protein-energy supplement*” or “protein and energy supplement*” or “protein calorie supplement*” or “protein-calorie supplement*” or “protein and calorie supplement*” or “oral nutrition*” or ((oral or orally) N10 nutrition* N10 supplement*) or ((oral or orally) and (“enteral nutrition*” or “enteral supplement*” or “enteral feeding*”) NOT (“tube feeding*” ))) ) AND ( AB (child* or schoolchild* or “school child*” or kid or kids or toddler* or adoles* or preadolescen* or pre-adolescen* or preteen* or teen* or boy* or girl* or minors or underage* or “under ag*” or juvenile* or youth* or kindergar* or puber* or pubescen* or prepubescent* or prepuberty* or pediatric* or paediatric* or peadiatric* or schools or “nursery school*” or preschool* or “pre school*” or “primary school*” or “secondary school*” or “elementary school*” or “high school*” or highschool* or “school age” or schoolage or “school age*” or schoolage* or student* or youngster*) OR TI (child* or schoolchild* or “school child*” or kid or kids or toddler* or adoles* or preadolescen* or pre-adolescen* or preteen* or teen* or boy* or girl* or minors or underage* or “under ag*” or juvenile* or youth* or kindergar* or puber* or pubescen* or prepubescent* or prepuberty* or pediatric* or paediatric* or peadiatric* or schools or “nursery school*” or preschool* or “pre school*” or “primary school*” or “secondary school*” or “elementary school*” or “high school*” or highschool* or “school age” or schoolage or “school age*” or schoolage* or student* or youngster*) ) AND ( AB (stunted or stunting or underweight or “under-weight” or wasting or wasted or “failure to thrive” or “growth retardation” or “growth faltering” or “growth failure” or “failure to grow” or “delayed growth*” or “restricted growth*” or “suboptimal growth*” or “sub-optimal growth*” or “catch-up growth*” or “catch-up growth*” or malnutrition or malnourishment or malnourished or “mal-nourished” or “under-nourished” or undernourishment or undernutrition or “under-nutrition” or undernourished or “picky eating” or “picky eater*” or “fussy eater*” or “feeding disorder*” or “infantile anorexia”) OR TI (stunted or stunting or underweight or “under-weight” or wasting or wasted or “failure to thrive” or “growth retardation” or “growth faltering” or “growth failure” or “failure to grow” or “delayed growth*” or “restricted growth*” or “suboptimal growth*” or “sub-optimal growth*” or “catch-up growth*” or “catch-up growth*” or malnutrition or malnourishment or malnourished or “mal-nourished” or “under-nourished” or undernourishment or undernutrition or “under-nutrition” or undernourished or “picky eating” or “picky eater*” or “fussy eater*” or “feeding disorder*” or “infantile anorexia”) )
Web of Science	Science Citation Index Expanded Social Sciences Citation Index Emerging Sources Citation Index	( AB = (“oral nutritional supplement*” or “oral nutrition supplement*” or “young child formula*” or PediaSure or Sustagen or NUTREN or “Boost Kid Essentials” or Kindercal or “Bright Beginnings” or “Peptamen Junior” or “S-26 PE Gold” or “Junior Horlicks” or Bournvita or Complan or Fortisip or Fortini or “Protinex Junior” or “Nutrilite Kids” or Dinoshake or “Nutraherbs Kids” or “Amul Pro” or Wyeth or “NG Solutions” or “Mead Johnson” or Nestle or Nutricia or ((nutrition or nutritional or nutrient or nutrients) NEAR/10 (“oral supplement*” or “dietary supplement*” or “diet supplement*”)) or “supplementary oral nutrition” or “supplemented oral nutrition” or “supplementary enteral nutrition” or “supplemented enteral nutrition” or “supplemental enteral nutrition” or ((oral or orally) and (“enteral formula*” or “enteric formula*” or “polymeric formula*” or “polymeric diet*”)) or “oral nutritional intervention*” or “sip feed*” or “nutrition drink” or “nutrition drinks” or “nutritional drink*” or “milk drink” or “milk drinks” or “fortified milk*” or “enriched milk*” or “fortified beverage*” or “liquid nutrition* supplement*” or “oral enteral nutrition*” or “oral enteric nutrition*” or “oral enteral feeding*” or “protein energy supplement*” or “protein-energy supplement*” or “protein and energy supplement*” or “protein calorie supplement*” or “protein-calorie supplement*” or “protein and calorie supplement*” or “oral nutrition*” or (((oral or orally) NEAR/10 nutrition*) NEAR/10 supplement*) or ((oral or orally) and (“enteral nutrition*” or “enteral supplement*” or “enteral feeding*”) NOT (“tube feeding*” ))) OR TI = (“oral nutritional supplement*” or “oral nutrition supplement*” or “young child formula*” or PediaSure or Sustagen or NUTREN or “Boost Kid Essentials” or Kindercal or “Bright Beginnings” or “Peptamen Junior” or “S-26 PE Gold” or “Junior Horlicks” or Bournvita or Complan or Fortisip or Fortini or “Protinex Junior” or “Nutrilite Kids” or Dinoshake or “Nutraherbs Kids” or “Amul Pro” or Wyeth or “NG Solutions” or “Mead Johnson” or Nestle or Nutricia or ((nutrition or nutritional or nutrient or nutrients) NEAR/10 (“oral supplement*” or “dietary supplement*” or “diet supplement*”)) or “supplementary oral nutrition” or “supplemented oral nutrition” or “supplementary enteral nutrition” or “supplemented enteral nutrition” or “supplemental enteral nutrition” or ((oral or orally) and (“enteral formula*” or “enteric formula*” or “polymeric formula*” or “polymeric diet*”)) or “oral nutritional intervention*” or “sip feed*” or “nutrition drink” or “nutrition drinks” or “nutritional drink*” or “milk drink” or “milk drinks” or “fortified milk*” or “enriched milk*” or “fortified beverage*” or “liquid nutrition* supplement*” or “oral enteral nutrition*” or “oral enteric nutrition*” or “oral enteral feeding*” or “protein energy supplement*” or “protein-energy supplement*” or “protein and energy supplement*” or “protein calorie supplement*” or “protein-calorie supplement*” or “protein and calorie supplement*” or “oral nutrition*” or (((oral or orally) NEAR/10 nutrition*) NEAR/10 supplement*) or ((oral or orally) and (“enteral nutrition*” or “enteral supplement*” or “enteral feeding*”) NOT (“tube feeding*” ))) ) AND ( AB = (child* or schoolchild* or “school child*” or kid or kids or toddler* or adoles* or preadolescen* or pre-adolescen* or preteen* or teen* or boy* or girl* or minors or underage* or “under ag*” or juvenile* or youth* or kindergar* or puber* or pubescen* or prepubescent* or prepuberty* or pediatric* or paediatric* or peadiatric* or schools or “nursery school*” or preschool* or “pre school*” or “primary school*” or “secondary school*” or “elementary school*” or “high school*” or highschool* or “school age” or schoolage or “school age*” or schoolage* or student* or youngster*) OR TI = (child* or schoolchild* or “school child*” or kid or kids or toddler* or adoles* or preadolescen* or pre-adolescen* or preteen* or teen* or boy* or girl* or minors or underage* or “under ag*” or juvenile* or youth* or kindergar* or puber* or pubescen* or prepubescent* or prepuberty* or pediatric* or paediatric* or peadiatric* or schools or “nursery school*” or preschool* or “pre school*” or “primary school*” or “secondary school*” or “elementary school*” or “high school*” or highschool* or “school age” or schoolage or “school age*” or schoolage* or student* or youngster*) ) AND ( AB = (stunted or stunting or underweight or “under-weight” or wasting or wasted or “failure to thrive” or “growth retardation” or “growth faltering” or “growth failure” or “failure to grow” or “delayed growth*” or “restricted growth*” or “suboptimal growth*” or “sub-optimal growth*” or “catch-up growth*” or “catch-up growth*” or malnutrition or malnourishment or malnourished or “mal-nourished” or “under-nourished” or undernourishment or undernutrition or “under-nutrition” or undernourished or “picky eating” or “picky eater*” or “fussy eater*” or “feeding disorder*” or “infantile anorexia”) OR TI = (stunted or stunting or underweight or “under-weight” or wasting or wasted or “failure to thrive” or “growth retardation” or “growth faltering” or “growth failure” or “failure to grow” or “delayed growth*” or “restricted growth*” or “suboptimal growth*” or “sub-optimal growth*” or “catch-up growth*” or “catch-up growth*” or malnutrition or malnourishment or malnourished or “mal-nourished” or “under-nourished” or undernourishment or undernutrition or “under-nutrition” or undernourished or “picky eating” or “picky eater*” or “fussy eater*” or “feeding disorder*” or “infantile anorexia”) )
Scopus	Scopus	TITLE-ABS-KEY( (“oral nutritional supplement*” or “oral nutrition supplement*” or “young child formula*” or PediaSure or Sustagen or NUTREN or “Boost Kid Essentials” or Kindercal or “Bright Beginnings” or “Peptamen Junior” or “S-26 PE Gold” or “Junior Horlicks” or Bournvita or Complan or Fortisip or Fortini or “Protinex Junior” or “Nutrilite Kids” or Dinoshake or “Nutraherbs Kids” or “Amul Pro” or Wyeth or “NG Solutions” or “Mead Johnson” or Nestle or Nutricia or ((nutrition or nutritional or nutrient or nutrients) W/10 (“oral supplement*” or “dietary supplement*” or “diet supplement*”)) or “supplementary oral nutrition” or “supplemented oral nutrition” or “supplementary enteral nutrition” or “supplemented enteral nutrition” or “supplemental enteral nutrition” or ((oral or orally) and (“enteral formula*” or “enteric formula*” or “polymeric formula*” or “polymeric diet*”)) or “oral nutritional intervention*” or “sip feed*” or “nutrition drink” or “nutrition drinks” or “nutritional drink*” or “milk drink” or “milk drinks” or “fortified milk*” or “enriched milk*” or “fortified beverage*” or “liquid nutrition* supplement*” or “oral enteral nutrition*” or “oral enteric nutrition*” or “oral enteral feeding*” or “protein energy supplement*” or “protein-energy supplement*” or “protein and energy supplement*” or “protein calorie supplement*” or “protein-calorie supplement*” or “protein and calorie supplement*” or “oral nutrition*” or (((oral or orally) W/10 nutrition*) W/10 supplement*) or ((oral or orally) and (“enteral nutrition*” or “enteral supplement*” or “enteral feeding*”) AND NOT (“tube feeding*” ))) AND (child* or schoolchild* or “school child*” or kid or kids or toddler* or adoles* or preadolescen* or pre-adolescen* or preteen* or teen* or boy* or girl* or minors or underage* or “under ag*” or juvenile* or youth* or kindergar* or puber* or pubescen* or prepubescent* or prepuberty* or pediatric* or paediatric* or peadiatric* or schools or “nursery school*” or preschool* or “pre school*” or “primary school*” or “secondary school*” or “elementary school*” or “high school*” or highschool* or “school age” or schoolage or “school age*” or schoolage* or student* or youngster*) AND (stunted or stunting or underweight or “under-weight” or wasting or wasted or “failure to thrive” or “growth retardation” or “growth faltering” or “growth failure” or “failure to grow” or “delayed growth*” or “restricted growth*” or “suboptimal growth*” or “sub-optimal growth*” or “catch-up growth*” or “catch-up growth*” or malnutrition or malnourishment or malnourished or “mal-nourished” or “under-nourished” or undernourishment or undernutrition or “under-nutrition” or undernourished or “picky eating” or “picky eater*” or “fussy eater*” or “feeding disorder*” or “infantile anorexia”)

* denotes a wildcard.
